# GDF11: An emerging therapeutic target for liver diseases and fibrosis

**DOI:** 10.1111/jcmm.18140

**Published:** 2024-03-17

**Authors:** Pardis Habibi, Kimia Falamarzi, Niloofar Dehdari Ebrahimi, Mohammad Zarei, Mahdi Malekpour, Negar Azarpira

**Affiliations:** ^1^ Student Research Committee Shiraz University of Medical Sciences Shiraz Iran; ^2^ Transplant Research Center Shiraz University of Medical Sciences Shiraz Iran; ^3^ Renal Division, Brigham & Women's Hospital Harvard Medical School Boston Massachusetts USA; ^4^ John B. Little Center for Radiation Sciences Harvard T.H. Chan School of Public Health Boston Massachusetts USA

**Keywords:** GDF11, growth differentiation factor 11, HCC, MAFLD, NAFLD

## Abstract

Growth differentiation factor 11 (GDF11), also known as bone morphogenetic protein 11 (BMP11), has been identified as a key player in various biological processes, including embryonic development, aging, metabolic disorders and cancers. GDF11 has also emerged as a critical component in liver development, injury and fibrosis. However, the effects of GDF11 on liver physiology and pathology have been a subject of debate among researchers due to conflicting reported outcomes. While some studies suggest that GDF11 has anti‐aging properties, others have documented its senescence‐inducing effects. Similarly, while GDF11 has been implicated in exacerbating liver injury, it has also been shown to have the potential to reduce liver fibrosis. In this narrative review, we present a comprehensive report of recent evidence elucidating the diverse roles of GDF11 in liver development, hepatic injury, regeneration and associated diseases such as non‐alcoholic fatty liver disease (NAFLD), non‐alcoholic steatohepatitis (NASH), liver fibrosis and hepatocellular carcinoma. We also explore the therapeutic potential of GDF11 in managing various liver pathologies.

## 1 BACKGROUND

The liver is a vital organ that plays an essential role in various functions within the body, including metabolism, food digestion, detoxification and vitamin storage.^1^ It is subject to various chronic diseases caused by metabolic syndromes, obesity, excessive alcohol consumption, viral hepatitis and other chronic conditions. These diseases can lead to liver fibrosis, steatohepatitis, liver cirrhosis and hepatocellular carcinoma (HCC).^2^, ^3^ Globally, liver diseases cause two million deaths each year and can result in severe disabilities.^4^ Metabolic disorders can induce fatty liver diseases. Approximately 25% of people worldwide have metabolic‐associated fatty liver disease (MAFLD).

Despite the importance and burden of liver diseases, the main ways to prevent chronic liver diseases (CLDs) are preventive approaches such as reducing weight, proper vaccination against hepatitis viruses and decreasing alcohol consumption.^5^ Although studies have discovered new therapeutic targets for curing CLDs, enough proper therapeutics have not yet been discovered. Growth differentiation factor 11 (GDF11) protein is one of the emerging targets with beneficial effects on reducing liver fibrosis and cirrhosis.^3^, ^6^, ^7^

GDF11, also known as bone morphogenetic protein 11 (BMP11), is a member of the transforming growth factor‐β (TGF‐β) family.^8^ Recent studies have demonstrated that recombinant GDF11 is an effective anti‐aging agent for the heart, brain and muscles.^9^ Additionally, GDF11 has an impact on liver development^10^, ^11^ and liver‐associated diseases like MAFLD and NAFLD,^7^, ^12^ HCC^13^ and liver fibrosis.^7^

In previous studies, GDF11 was shown to have controversial effects on liver fibrosis and cirrhosis.^14^ While some studies indicated that GDF11 could impair liver regeneration^15^ and exacerbate liver injury,^16^ others found that GDF11 could inhibit liver fibrosis^7^, ^17^ and might protect against HCC.^13^ These controversies could be due to misunderstandings caused by the complexity of biological systems and the different roles of GDF11.^18^ In this study, we aim to explain the molecular effects of GDF11 and its roles in liver‐associated diseases and the possibility of utilizing GDF11 as a drug target for these diseases.

### 1.1 The physiological roles of GDF11 in the body

GDF11, is a part of BMP family that is a subfamily of the TGF‐β superfamily that secretes important signalling components essential for human development.^19^ In humans, the GDF11 gene is located on chromosome 12q13.2 and encodes a protein with 407 amino acids that is inactive after transcription.^9^ The GDF11 protein must be cleaved by the pro‐protein convertase subtilisin/kexin type 5 (PCSK5), a signal peptidase, and then activated by the BMP1/Tolloid family of metalloproteases.^9^ As a secretory protein, GDF11 is expressed in most human body tissues, including the liver, kidneys, heart, pancreas, intestines, skeletal muscles and nervous system.^20^, ^21^ GDF11 induces the recruitment of activin receptor I (ActRI) by binding to activin receptor II (ActRII), leading to the formation of a complex that activates the downstream pathway with kinase functions.^9^ The GDF11‐ActR complex activates various molecules, mainly consisting of suppressors of mothers against decapentaplegic (SMAD) and non‐SMAD pathways. SMAD proteins can translocate to the nucleus and transduce signalling as transcription factors.^22^ SMAD mediators play crucial roles in the TGF‐β signalling pathway, which is tissue‐specific. TGF‐β signalling can regulate the pluripotency of stem cells during embryonic development via BMP pathways and SMAD mediators.^23^ Non‐SMAD proteins are also various components that can be affected by GDF11 like mitogen‐activated protein kinase (MAPK), phosphatidylinositol 3 kinase (PI3K)/AKT, and Rho‐like GTPase. Activation of non‐SMAD pathways can contribute to many biological processes such as regulating inflammation and cell nucleus size.^9^ GDF11 regulates gene expression in different tissues via activating SMAD and non‐SMAD pathways.^9^

**FIGURE 1:**
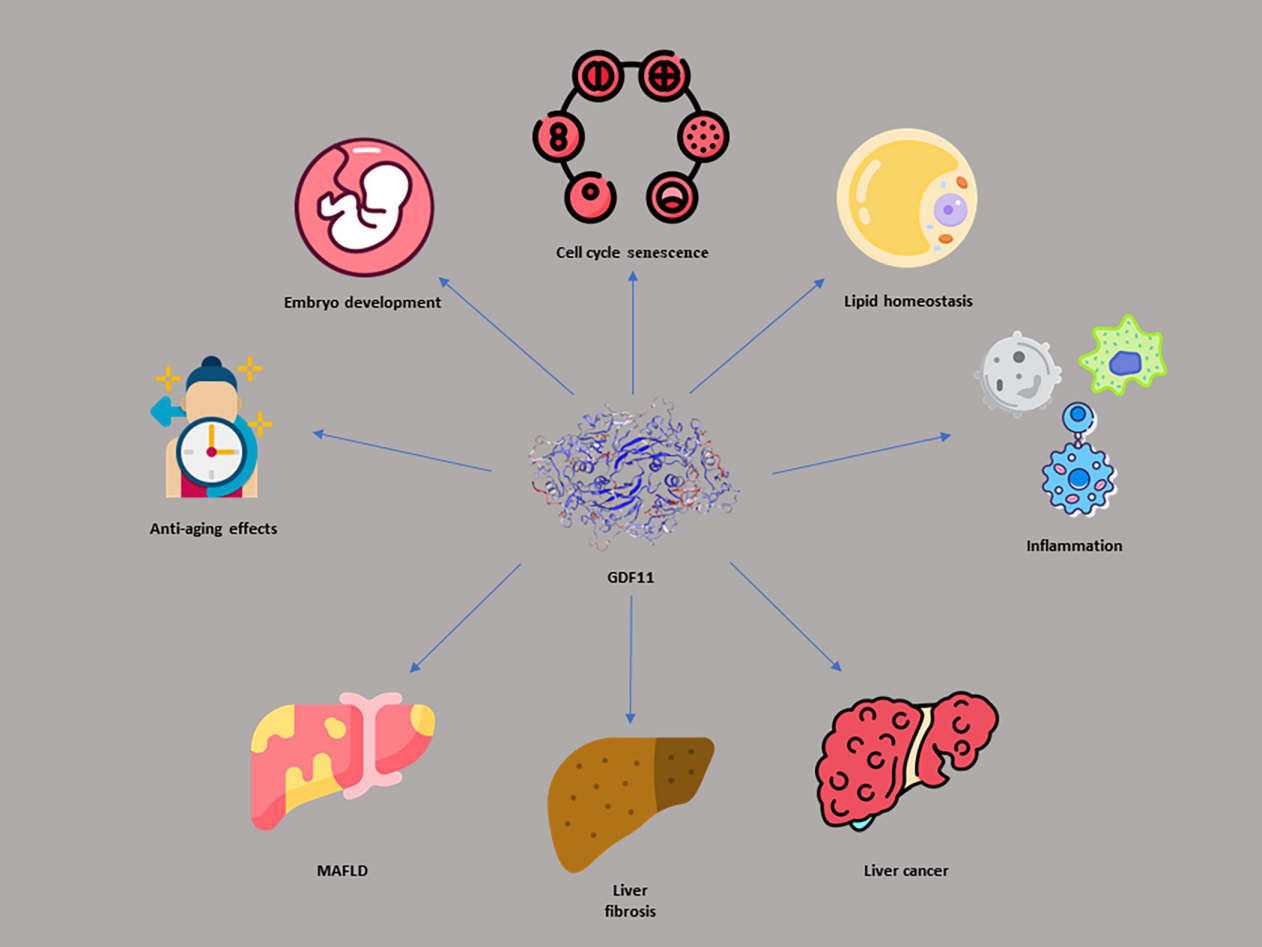
The contribution of GDF11 in different aspects of the pathophysiology of the human body.

GDF11 affects various biological functions including reducing inflammation,^24^ lipid homeostasis,^25^ angiogenesis^26^ and anti‐fibrotic properties.^14^ Moreover, GDF11 participates in the pathogenesis of various disorders such as diabetes mellitus,^27^ cardiovascular diseases,^28^ metabolic disorders,^29^ cancer^18^ and aging.^30^ Figure 1 illustrates some of the physiopathologic roles of GDF11 in the body.

GDF11 can be regarded as an anti‐aging factor with anti‐fibrotic properties.^14^, ^30^ In different organs including the skin, neurological system, and heart, GDF11 exhibits anti‐aging and regenerative properties. Its anti‐aging effects may arise from features such as neurogenic and angiogenic features.^31^ Additionally, GDF11 can induce rejuvenation processes in tissues by stimulating stem cells.^32^ On the other hand, GDF11 may influence the stemness capacity of some cancer cells thereby increasing their aggressiveness.^18^

Several studies have reported substantial roles of GDF11 in regulating lipid and glucose homeostasis.^21^, ^27^, ^33^ GDF11 maintains insulin secretion, β‐cell function, and survival in mouse models of Type 2 diabetes (T2D).^27^ GDF11 inhibits glucose‐induced β‐cell apoptosis and decreases glucagon secretion by activating TGF‐β/SMAD2 and PI3K‐AKT‐B‐forkhead box protein O1 (PI3K‐AKT‐FoxO1) pathways, resulting in enhanced glucose homeostasis.^21^ Furthermore, GDF11 inhibits adipogenesis via phosphorylation of SMAD2/3 and activation of the wingless‐related integration site (WNT)/β‐catenin pathway in pre‐adipocytes. It also inhibits adipogenesis by increasing adiponectin expression and glucose uptake in mature adipocytes by affecting the activin receptor‐like kinase 5 (ALK5)‐SMAD2/3 signalling pathway.^34^ Consequently, GDF11 improves glucose and insulin homeostasis and reduces weight gain and white adipocyte (WAT) size.^34^ Walker et al. reported that administration of GDF11 reduces weight gain and improves glucose tolerance in aged mice on a high‐fat diet (HFD).^33^ However, the precise mechanism for this enhancement in glucose uptake has not been determined but it was not due to an increase in β‐cell proliferation or insulin secretion.^33^ Therefore, further investigations are needed to discover the exact roles of GDF11 in glucose and lipid metabolism.

GDF11 is also capable of serving as a therapeutic agent. Recombinant GDF11 has been used to rejuvenate different organs including the heart, brain, and skeletal muscles. In this process, different results were observed and different explanations were offered to explain them. Various reasons were proposed including the diversity of folding and refolding of GDF11, the variety of sources and quality of recombinant GDF11, and the concentrations of GDF11 used.^35^

## 2 GDF11 IN LIVER DEVELOPMENT, SENESCENCE AND REGENERATION

### 2.1 GDF11 in liver development

GDF11 plays an extensive role in embryonic development. It participates in anterior/posterior patterning of the spinal cord, development of skeletal muscle, bone, nervous system and the urogenital system as well as digestive system glands, retinal development, and erythropoiesis.^9^, ^36^, ^37^, ^38^, ^39^ GDF11 has inhibitory effects on zebrafish liver development since its overexpression resulted in a smaller liver phenotype, probably due to inhibition of hepatocyte proliferation.^9^, ^11^ GDF11 is a target of histone deacetylase 3 (HDAC3), an essential factor for GDF11 promoter inactivation. HDAC3 inhibition leads to GDF11 upregulation.^40^ HDAC3 knockout mice showed defects in liver regeneration and hepatocyte proliferation arrest in the G1 phase of the cell cycle.^41^ Although to the best of our knowledge, GDF11's role in liver formation has not been studied in mammals, previous studies on other species (zebrafish) and different cell lines suggested that GDF11 exerted its effects on liver development through direct action on hepatocyte precursor (hepatoblast) proliferation.^11^, ^13^, ^18^, ^40^ Therefore, further investigations are necessary to determine the precise roles of GDF11 in inhibiting liver development and hepatocyte proliferation.

A recent study revealed that serum and hepatic expression of GDF11 increased in mouse models of partial hepatectomy. Moreover, treatment with recombinant GDF11 and adeno‐associated viruses‐GDF11 led to severe impairment of liver regeneration while suppressing GDF11 activity‐ameliorated liver regeneration.^15^ Accordingly, GDF11 induced cell cycle arrest and suppressed liver cell proliferation via activating the TGF‐β‐SMAD2/3 signalling pathway resulting in impairment of liver regeneration.^15^

### 2.2 GDF11 in liver senescence

GDF11 also contributes to the senescence process.^10^ Senescence is a biological process that shows the permanent arrest of the cell cycle and is associated with regeneration disruption, inflammation, and tumorigenesis.^42^ Aging is accompanied by decreased cellular autophagy which leads to the accumulation of damaged intracellular components and thereby disruption of homeostasis.^43^ Autophagy is an intracellular homeostatic process that results in lysosome‐dependent degradation of intracellular components.^44^

According to Sun et al., GDF11 may downregulate liver cell autophagy by inducing the mammalian target of rapamycin complex 1 (mTORC1).^10^ mTORC1 is an intracellular component that suppresses autophagy via inhibiting transcription factor EB (TFEB).^45^ Overexpression of GDF11 in aged mice resulted in increased expression of p16, one of the indicators of cellular senescence in hepatic cells.^10^ GDF11 indirectly decreased autophagosome‐lysosome fusion, lysosome production and lysosome proteolytic activity resulting in increased cellular senescence.^10^ Also, knockdown of GDF11 decreased cellular senescence and increased liver proliferation due to increased autophagic flux.^10^

### 2.3 GDF11 in liver ischaemia–reperfusion injury

Ischaemia–reperfusion injury (IRI) is a complicated process that occurs during liver transplantation, trauma, shock and hepatic surgery.^46^, ^47^ IRI occurs when damage caused by hypoxia and ischaemia worsens after blood flow restoration and oxygen delivery.^48^, ^49^ Mitochondrial impairment, calcium overload, oxidative stress, Kupffer cell and neutrophil activation and proinflammatory cytokine release are among the processes implicated in hepatic IRI.^50^, ^51^, ^52^, ^53^ Serum and hepatic levels of GDF11 elevated in liver IRI in both young and aged mice. Also, administration of recombinant GDF11 resulted in aggravation of liver histologic status and liver regeneration defect.^16^ Cyclin D1, cyclin E1, cyclin‐dependent kinase 4 (CDK4) and CDK2 accelerate cell cycle progression from G1 to the S‐phase. GDF11 could downregulate these factors by increasing p16 (inhibitor of CDK4/6) and p27Kip1 (inhibitor of CDK2) thus suppressing hepatocyte regeneration.^16^ Zhang et al. discovered that GDF11 improved recovery of tubular regeneration and renal function after kidney IRI in elderly mice.^54^ Also, another study performed by Du et al. showed that targeted delivery of the GDF11 gene to the myocardium rejuvenated the aged mouse heart and improved myocardial regeneration after IRI.^55^ These studies demonstrated protective effects of GDF11 in the kidney and heart after IRI in contrast to its roles in liver IRI suggesting that GDF11 expression and function may be tissue‐specific.^16^, ^54^, ^55^

## 3 GDF11 IN LIVER‐ASSOCIATED DISEASES

### 3.1 GDF11 in NAFLD and MAFLD

NAFLD is one of the world's major causes of liver disease and affects about one‐quarter of the world's population.^56^ NAFLD is characterized by fat accumulation in more than 5% of liver weight, while other liver diseases, for example, drug‐induced liver injury, excessive alcohol intake and viral hepatitis, are excluded.^56^, ^57^ NAFLD is found to be associated with cardiovascular and metabolic disorders, such as obesity, hypertension, insulin resistance (IR), T2D and dyslipidemia.^58^ Due to the association of NAFLD with metabolic diseases, NAFLD can be counted as the hepatic indicator of metabolic syndrome.^58^ Regarding the strong link between NAFLD and metabolic syndrome, experts have established a new term, MAFLD, defined by the presence of hepatic steatosis with confirmed T2D, obesity, or metabolic dysregulation.^58^ To attribute metabolic dysregulation to someone, at least two metabolic abnormalities are required, including increased waist circumference, prediabetes, abnormal plasma triglycerides (TG), high‐density lipoprotein cholesterol (HDL), blood pressure, high‐sensitivity C‐reactive protein (hs‐CRP), and homeostasis model assessment of insulin resistance (HOMA‐IR).^58^

During NAFLD, hepatic steatosis occurs due to an imbalance in lipid input and export.^59^ Increased dietary intake, decreased mitochondrial fatty acid oxidation and decreased hepatic lipid export can result in hepatic steatosis.^59^ Some genetic variants, for example missense variants in transmembrane 6 superfamily member 2 (*TM6SF2*) and in patatin phospholipase‐like domain containing protein 3 (*PNPLA3*) are associated with lipid imbalance resulting in inducing hepatic steatosis.^60^, ^61^ Moreover, inflammation occurs during hepatic steatosis, which can lead to NAFLD progression.^62^ NAFLD might also progress to non‐alcoholic steatohepatitis (NASH), characterized by evidence of hepatocyte injury with histological findings of steatohepatitis.^56^ Individuals suffering from NASH are at an increased risk of cardiovascular diseases and HCC. Chronicity of hepatocytes injury during NASH might eventually cause fibrosis and cirrhosis.^63^ Nowadays, NASH is among the leading causes of liver cirrhosis and is the second cause of liver transplantation in the United States.^56^, ^64^ Because MAFLD and NASH are highly burdensome, much effort has been focused on understanding their pathophysiological mechanisms.^65^ In this regard, GDF11 is one of the many molecules that can interfere with pathways involved in the pathogenesis of NAFLD.^12^, ^65^

Dai et al. discovered a higher expression of GDF11 in patients with NAFLD than in the normal population.^7^ On the other hand, GDF11 expression was not altered in mice fed a HFD.^7^ Hence, Dai et al. hypothesized that GDF11 would only increase in response to fibrosis, and in the case of HFD mice, there was neither an increase in fibrosis nor an increase in GDF11.^7^ GDF11 treatment of the genetically modified (ob/ob) mice neither resulted in overt hepatic lipid accumulation nor increased liver inflammation and injury. GDF11 administration in ob/ob mice could only increase activated hepatocyte stellate cells (HSCs) via influencing ALK5/SMAD2/3/AKT‐dependent signalling pathways and might lead to mild profibrogenic effects in vivo; yet, GDF11 administration didn't result in an increased liver inflammation or injury. A decrease in Kupffer cell number (with an inflammatory role) was also achieved after GDF11 treatment of ob/ob mice.^12^ In summary, these data indicated that GDF11 may trigger the first stages of NASH development and fibrosis by HSCs activation and deposition of extracellular matrix (ECM) components in the absence of liver injury and inflammation.^12^ Additionally, Frohlich et al. demonstrated a considerably positive correlation between GDF11 mRNA levels and expression of genes that participated in NAFLD progression like peroxisome proliferator‐activated receptor gamma (PPARγ) and carnitine palmitoyl transferase 1 (CPT1).^12^

On the contrary, Xu et al. found that decreased levels of GDF11 have been associated with NAFLD, increased body mass index (BMI), increased diastolic blood pressure, TG, low‐density lipoprotein (LDL), HOMA‐IR, fasting blood sugar (FBS), and two‐hour postprandial glucose (2hpp), as well as metabolic syndrome‐related morbidity in Chinese population.^29^ GDF11 exhibited a stronger inverse relationship with the metabolic profile in men.^29^ GDF11 administration in HFD mice significantly reduced NAFLD activity score (NAS), FBS, insulin level, lipid content and the expression of some genes involved in gluconeogenesis.^7^ A mild but significant decrease in body weight was also observed using GDF11 in HFD mice.^7^ Together, these data indicate that overexpression of GDF11 prevents the development of NASH in HFD mice.^7^ Another study on HFD mice showed that GDF11 administration increased glucose tolerance, reduced food intake, and prevented weight gain in HFD mice, but GDF11 could not inhibit age‐ or HFD‐induced hepatosteatosis despite the improvement observed in other metabolic parameters.^33^ Although GDF11 involves in pancreatic development and β‐cells survival, an improvement in glucose tolerance following GDF11 treatment wasn't associated with pancreatic β‐cell replication or increased insulin secretion.^27^, ^33^, ^66^, ^67^

In addition to improved glucose tolerance, insulin resistance, gluconeogenesis reduction, and weight gain inhibition, GDF11 administration decreased the number and size of lipid droplets in the hepatocytes of HFD mice and resulted in hepatic steatosis reduction.^21^ In HFD mice, GDF11 administration contributed to the recruitment of lipids in brown adipose tissue (BAT).^21^ GDF11 also increased proteins in charge of energy expenditure and thermogenesis in BAT of the HFD mice, resulting in a 0.4°C increase in rectal temperature.^21^ Furthermore, GDF11 prevented inflammation and macrophage infiltration in the epididymal white adipose tissue (WAT) of the HFD mice.^21^ In HFD mice, expression of the genes involved in fatty acid β‐oxidation was not altered after GDF11 treatment.^21^ One possibility was that GDF11 reduced hepatocyte fat intake by altering lipid distribution and entering the lipids into the thermogenesis pathway.^21^ Another possibility was that GDF11 reduced metabolic disorders by decreasing inflammation, which is responsible for many metabolic complications such as obesity.^21^ Therefore, GDF11 increased thermogenesis, energy expenditure, and regulated glucose and lipid metabolism through TGF‐β/SMAD2, PI3K/AKT/FoxO1 and AMPK pathways, which might eventually lead to the prevention of obesity and other metabolic disorders.^21^

The summary of the effects of GDF11 on the NAFLD models can be seen in Table 1. Regarding the impact of the GDF11 molecule in various metabolic pathways GDF11 is a promising target for the prevention and treatment of MAFLD and other metabolic disorders in the future. However, there are several inconsistencies among the results of previous investigations; Hence, more research is required to determine the different aspects of its therapeutic and potentially harmful effects.

**TABLE 1:** GDF11 effects on the NAFLD models.

GDF11 analogue	Species	Hepatic fat content/inflammation	Hepatic fibrosis	Glycaemic control	Serum lipid profile	Body weight	References
AAV.GDF11	HFD‐fed mice	Decrease NAS score and lipid content	Decrease liver fibrosis	Decrease fasting glucose and insulin levels	‐	Decrease body weight	^7^
rGDF11	HFD‐fed mice	No changes in hepatosteatosis	‐	Improved GTT, reduced blood glucose	‐	Decrease body weight	^33^
rGDF11	Ob/ob mice	No changes in lipid accumulation, no induction of liver injury or inflammation	Activated HSC cells, triggered liver fibrosis	‐	‐	Decrease body weight	^12^
Plasmid carrying GDF11	HFD‐fed mice	Suppressed hepatic fat accumulation	‐	Decreased fasting glucose and insulin level, improved GTT and insulin sensitivity	Decreased serum TG and total cholesterol	Prevented weight gain	^21^

### 3.2 GDF11 in liver fibrosis

Liver fibrosis, with a prevalence of 0.7%–25.7%, is one of the leading causes of global morbidity and mortality. Untreated fibrosis can result in cirrhosis and other complications including HCC.^68^, ^69^, ^70^ Even though liver fibrosis is reversible, there is currently no confirmed treatment for it.^71^, ^72^, ^73^ Therefore, understanding the pathogenesis of liver fibrosis and finding possible diagnostic and therapeutic markers is essential.^73^ Excessive alcohol consumption, viral infections, and MAFLD are among the leading causes of hepatic fibrosis.^14^

Liver fibrosis is thought to be caused by the interaction of various cells including hepatocytes, HSCs, and Kupffer cells as well as the infiltration of immune cells.^74^ As a result of persistent and chronic liver injury, profibrotic and inflammatory cytokines increase which cause the activation of HSCs. Activated HSCs can transform into myofibroblasts which enhance the production and deposition of extracellular matrix components resulting in fibrosis.^14^, ^75^ Chronic hepatic injury, inflammatory cytokine release, recruitment of inflammatory cells, and excessive ECM production (mainly collagen type I) are critical mechanisms in liver fibrosis.^73^ Additionally, chronic liver injury is associated with the proliferation of leucine‐rich repeat‐containing G‐protein‐coupled receptor 5+ (LGR5+) liver progenitor cells (LPCs) which are absent in normal livers. These LGR5+ LPCs can attenuate liver fibrosis.^7^, ^76^

Administration of GDF11, which is elevated in human and mice fibrotic livers, enhanced the expansion of LGR5+ cells in mouse and human liver organoids.^7^ In mouse models of hepatic fibrosis, LGR5+ LPCs treated with GDF11 suppressed fibrogenesis.^7^, ^76^ Overexpression of GDF11 in hepatic myofibroblasts may act as a growth factor to preserve LGR5+ progenitor proliferation.^7^ Following transplantation of LGR5+ cells into the mice spleen, expanded LGR5+ cells reversed activated HSCs to quiescent HSC phenotype cells by downregulating actin alpha 2 (ACTA2) (a fibrogenic gene and marker of activated HSCs).^7^ This process may explain the underlying mechanism through which GDF11‐induced LGR5+ cells decrease liver fibrosis.^7^ Another possibility about the beneficial effects of GDF11 in liver injury is that a GDF11‐enriched environment might promote the secretion of chemokines from progenitor cells resulting in scar‐resolving immune subset recruitment.^76^, ^77^, ^78^ Contrary to these results, Frohlich et al. showed that in obese (ob/ob) or lean wild‐type mice GDF11 enhanced profibrogenic program in HSCs worsened collagen deposition and increased α smooth muscle actin (αSMA) staining in the liver without affecting hepatic damage or inflammation.^12^ The observed controversy regarding outcomes might result from different models employed in‐vitro and in‐vivo investigation procedures and route and dosage of GDF11 administration.^14^ Consequently, further investigations are required to determine the exact mechanism and potential therapeutic effects of GDF11 in liver fibrosis.

### 3.3 GDF11 and liver cancer

HCC is the most common primary liver cancer and the third most common cancer in terms of mortality.^79^, ^80^ Despite the advances in recognition of HCC and its aetiologies, 5‐year survival rate of this cancer is only 21%.^81^ Hepatitis C virus (HCV) infection, hepatitis B virus (HBV) infection, alcoholic cirrhosis, aflatoxin‐contaminated food consumption, NASH, metabolic disorders and MAFLD are among the significant risk factors of HCC.^82^, ^83^ The risk factors of HCC usually contribute to liver injury by inducing chronic liver inflammation. The chronicity of liver injuries results in liver cirrhosis which contains focal areas of immature and abnormal hepatocytes; consequently, these dysplastic precancerous areas might develop HCC.^82^ In some cases, HCC can be generated in a non‐cirrhotic liver. Non‐cirrhotic HCC is more prevalent among HCC patients with NAFLD bases.^46^ Due to the late diagnosis of NAFLD‐induced HCC, prognostic outcomes are usually worse.^84^ Therefore, understanding the molecular mechanisms contributing to HCC development can help manage HCC better. GDF11 is suggested to be a regulator of differentiation in cells that retain stemness capacity, like HCC cells, so these cells are potential targets for GDF11.^13^, ^18^ Previously, the downregulation of GDF11 expression was discovered in patients with HCC.^85^ The altered expression of GDF11 in HCC models suggested that GDF11 might contribute to HCC development.^86^ Also, genetic mutations in TGF‐β core genes, including GDF11, play an important role in HCC pathogenesis.^87^, ^88^ Previous studies discovered tumour suppressive roles of GDF11 in HCC by reducing HCC cell proliferation, clonogenic capacity, cellular function, and aggressiveness, as well as causing dysregulation of cancer cell metabolism.^13^, ^18^, ^85^, ^89^

Gerardo‐Ramírez et al. found that GDF11 inhibits tumour progression via suppression of cellular proliferation and doesn't affect HCC cell viability.^13^ Additionally, cell proliferation of tumour cells was also inhibited by GDF11 in other studies.^89^, ^90^ Another study mentioned a reduction in HCC cells' viability in addition to proliferation inhibition after GDF11 treatment.^85^ GDF11 increased HCC cells' sensitivity to cisplatin.^91^ Tumour aggressiveness, stemness markers, and mesenchymal markers of HCC cells also decreased after GDF11 treatment in Gerardo‐Ramírez's study.^13^ Also, epithelial markers and cell‐to‐cell connections increased after GDF11 treatment of the HCC cells.^13^ As a result, GDF11 made a cytostatic condition and caused a decrement in epithelial to mesenchymal transition, resulting in decreased tumour aggressiveness and invasion.^13^

Frohlich et al. declared that treatment of HCC cells with GDF11 resulted in increased expression of genes that participated in fatty acid β‐oxidation lipid droplet formation and growth regulating fatty acid storage and steatosis.^90^ More lipid droplets with larger sizes were found in HCC cells. So an increase in lipid synthesis and a decrease in cell apoptosis were observed during GDF11 treatment. Whereas GDF11 reduced HCC cell apoptosis and served them with lipid energy GDF11 reduced tumour progression by inhibiting cell proliferation.^90^

On the contrary, in Sharik Hernandez et al.'s study, GDF11 administration in HCC cells impaired both glycolysis and oxidative phosphorylation.^89^ GDF11 suppressed the expression of genes involved in lipogenesis and the AKT/mammalian target of the rapamycin (mTOR) pathway which participates in lipid homeostasis in HCC cells.^89^ Both neutral lipids and cholesterol content of the HCC cells were diminished and changes in their mitochondria such as disarrangement of cristae partial cristolysis electron‐lucent matrix and reduced mitochondria size were observed. Results from this study indicated that GDF11 caused impairment of cancer cell lipid and glucose metabolism and induced defects in mitochondrial functions. Therefore, the tumour suppressive property of GDF11 was concomitant to the decrease in cholesterol transport activity and cholesterol homeostasis.^89^

Tumour‐associated macrophages are macrophages that reside in the tumour microenvironment.^92^ These macrophages predominantly shift to the M2 type in the HCC microenvironment and due to their immune‐suppressive phenotype are related to poorer prognosis of cancers.^93^ GDF11 treatment of the THP‐1‐macrophage cell line in the HCC model resulted in an increase in polarization of macrophages to M1 macrophages. It was concluded that GDF11 treatment of HCC might inhibit tumour progression by inducing polarization of macrophages to M1.^94^

An additional type of primary liver cancer cholangiocarcinoma arises from the epithelium of intra‐ or extrahepatic bile ducts.^95^, ^96^ The incidence of cholangiocarcinoma is less than 6 cases per 100,000 people. Cholangiocarcinoma is a rare but highly fatal cancer and its incidence rate has increased worldwide during the past decade.^96^ Cholangiocarcinoma is mostly asymptomatic at early stages and thus is diagnosed at advanced stages leading to poor prognosis and a 5‐year survival of 7%–20%.^97^, ^98^ Hence, finding new biomarkers for diagnostic and therapeutic means is of great importance.

A recent study has shown upregulation of PCSK5 and GDF11 in cholangiocarcinoma patients.^13^ Exosomes of patients with cholangiocarcinoma exhibited elevated miR‐3124‐5p levels which contribute to cancer cell proliferation migration and angiogenesis through downregulation of GDF11 expression. Consequently, downregulation of GDF11 may be responsible for enhancing cholangiocarcinoma aggression.^99^

Table 2 summarizes the effects of GDF11 on cancers of the liver. Based on the results of the above investigations targeting GDF11 might represent a therapeutic option in the future for liver cancers. Therefore, more investigation is needed to elucidate the exact function of GDF11 in HCC.

**TABLE 2:** Effects of GDF11 on liver cancer cells.

GDF11 analogue	Species	Effects on tumorigenesis	Effects on cancer cell metabolism	References
rGDF11	Human hepatic cancer cell lines	Decreased cell proliferation, no apoptosis induction	Increased lipid accumulation	^90^
rGDF11	Human HCC cell lines	Decreased cell proliferation	Decreased cholesterol content, impaired glycolysis	^89^
rGDF11	THP‐1‐macrophages cell line	No effects on cell proliferation, induce anti‐tumour macrophages response	Decreased lipid content	^94^
rGDF11	HCC cell lines	Decreased cell proliferation, decreased stemness and aggressiveness‐related genes	‐	^13^
rGDF11	HepG2 cell line	Decreased cell viability and inhibited proliferation	‐	^85^
rGDF11	HCC cell line	Increased sensitivity to chemotherapy treatment	‐	^91^
GDF11 downregulation	Serum exosomes of cholangiocarcinoma patients	Cholangiocarcinoma development	‐	^99^

## 4 THE ROLE OF GDF11 ANALOGUES AND LIVER‐ASSOCIATED DISEASES

Several studies have investigated the possible therapeutic roles of GDF11 in various pathological conditions including skeletal muscle injury, aging, stroke, and Alzheimer's disease.^35^, ^100^, ^101^, ^102^, ^103^ Moreover, the potential therapeutic effects of GDF11 on metabolic disorders such as obesity, T2D, glucose and insulin homeostasis dysregulation, and energy imbalance were recently evaluated.^21^, ^27^, ^33^ Additionally, GDF11 analogues play pivotal roles in liver‐associated diseases like NAFLD, HCC and liver fibrosis.^7^, ^12^, ^13^ However, there are inconsistencies in the impacts of GDF11 analogues on liver diseases which might be due to diversity in GDF11 dosage or the methods used for increasing GDF11 (such as recombinant GDF11 or GDF11 overexpression by plasmid or viral vectors).^33^, ^35^ Also, different protein refolding efficiencies, protein sources, and concentrations might result in different recombinant GDF11 concentrations and consequent disparities in study outcomes.^35^, ^104^

The promising effects of GDF11 analogues on metabolic disorders (such as obesity and T2D) and liver diseases like NAFLD, liver fibrosis, and HCC are still in early preclinical stages. Further studies are required to evaluate their efficacy and possible adverse effects.^35^ However, the use of GDF11 in clinics is questionable due to its low production efficiency, conflicting results about GDF11's rejuvenating effects, and the risk of inducing fibrosis.^18^, ^35^, ^104^ Table 3 summarizes the latest findings regarding the effects of GDF11 analogues on liver and metabolic disorders.

**TABLE 3:** The usage of GDF11 analogues in liver diseases.

GDF11 analogue	Diseases	Species	Results	References
rGDF11 AAV.GDF11	Liver fibrosis	Mouse and human liver organoids Mouse models of liver fibrosis	Attenuated liver fibrosis	^7^
rGDF11	NAFLD and liver fibrosis	ob/ob mouse models of obesity‐dependent NAFLD	Increased activated HSCs and liver fibrosis Reduced Kupffer cells and macrophages	^12^
rGDF11	Obesity	Human adipose‐derived stromal cells (HADSCs)	Inhibited adipogenic differentiation	^105^
Intraperitoneal injection of rGDF11	Metabolic disorders	Aged mice	Induced healthy weight loss	^106^
rGDF11	Metabolic disorders (Obesity)	ob/ob mice 3 T3‐L1 pre‐adipocytes 3T3‐L1 mature adipocytes	Decreased weight gain and WAT size Improved insulin sensitivity Inhibited adipogenesis Increased adiponectin levels and glucose uptake by mature 3 T3‐L1 adipocytes	^34^
rGDF11	HCC	Human hepatic cancer cell lines HepG2 and Hep3B	Enhanced lipogenic gene expression and lipid accumulation Decreased cell proliferation and apoptosis rate	^90^
rGDF11	HCC	Hepatocellular carcinoma‐derived cell lines	Impaired lipid metabolism, glycolysis, mitochondria function Decreased cells proliferation	^89^
rGDF11	HCC	HCC cell lines	Decreased cell proliferation Induced cell cycle arrest Decreased HCC cells invasion	^13^
rGDF11	Liver IRI	Mouse models of IRI	Worsened liver injury Enhanced liver regeneration impairment	^16^
rGDF11 AAV.GDF11	Liver injury	Mouse models of partial hepatectomy	Impaired liver regeneration	^15^
AAV.GDF11 Adenovirus‐small hairpin RNA‐GDF11	Liver senescence	Aged mice	Accelerated liver senescence	^10^

## 5 CONCLUSION

This study sheds light on the crucial role of GDF11 in liver development and pathophysiology of associated liver diseases including liver cancers and MAFLD. Despite limited knowledge about the role of GDF11 in liver development and diseases, its important role in the liver has been confirmed by many studies. In this study, we attempted to gather comprehensive knowledge about GDF11. However, further studies are needed to determine the exact role of GDF11 in liver‐associated diseases and its potential for use in targeted therapies.

## AUTHOR CONTRIBUTIONS

**Pardis Habibi:** Investigation (equal); writing – original draft (lead); writing – review and editing (equal). **Kimia Falamarzi:** Data curation (equal); writing – original draft (equal); writing – review and editing (equal). **Niloofar Dehdari Ebrahimi:** Data curation (equal); writing – original draft (equal); writing – review and editing (equal). **Mohammad Zarei:** Supervision (equal); writing – review and editing (equal). **Mahdi Malekpour:** Data curation (equal); writing – original draft (equal); writing – review and editing (equal). **Negar Azarpira:** Supervision (equal); writing – review and editing (equal).

## FUNDING INFORMATION

This work has no funding.

## CONFLICT OF INTEREST STATEMENT

The authors declare that the research was conducted in the absence of any commercial or financial relationships that could be construed as a potential conflict of interest.

## CONSENT FOR PUBLICATION

Not applicable.

## Data Availability

Data sharing is not applicable to this article as no datasets were generated or analysed during the current study.
